# Special Issue: Tissue Engineered Biomaterials and Drug Delivery Systems

**DOI:** 10.3390/pharmaceutics14122827

**Published:** 2022-12-16

**Authors:** Viviana P. Ribeiro, Joaquim M. Oliveira, Rui L. Reis

**Affiliations:** 1 3B’s Research Group, I3Bs—Research Institute on Biomaterials, Biodegradables and Biomimetics of University of Minho, Headquarters of the European Institute of Excellence on Tissue Engineering and Regenerative Medicine, AvePark, Parque de Ciência e Tecnologia, Zona Industrial da Gandra, Barco, 4805-017 Guimarães, Portugal; 2 ICVS/3B’s—PT Government Associate Laboratory, 4710-057 Braga, Portugal

Current advances in biomaterials processing and engineering for drug delivery have allowed interesting progressed in biomedical field. Such developments were only possible by the intervention of tissue engineers, physical and chemical scientists, biologists, and clinicians, that together developed innovative strategies and technologies capable of addressing a higher number and patient-specific requirements [1]. Biomaterials applied as anti-cancer drugs vehicles, and directed to cancer immunotherapy, neurodegenerative diseases, and genome editing are important achievements currently explored [2,3]. Responsive biomaterials used for cell targeting, intracellular drug delivery and gene therapy are highly attractive for precision medicine strategies directly applied to solve patients’ specific needs [4,5].

This Special Issue focus on the most recent tissue engineering strategies involving drug delivery systems for biomedical applications. For that, significant contributions addressing the above mentioned concepts were collected. This multidisciplinary topic resulted in the publication of nine original papers and two review papers, and are briefly summarized.

A novel encapsulation method of protein drugs into porous micro-scaffolds made of ammonium hydrogen carbonate (NH_4_HCO_3_) was proposed by Kang et al. [6]. An aqueous two-phase system (PEG/Sulfate) without denaturing conditions was applied to encapsulate α-Amylase into the porous micro-scaffolds. Pore-closed micro-scaffolds were successfully achieved, showing good integrity and activity for protein drug encapsulation and delivery.

Lamparelli et al. [7] proposed poly-lactic-co-glycolic acid microcarriers (PLGA-MCs) carrying a human Transforming Growth Factor β1 (hTFG-β1) to functionalize a collagen matrix used to create a three-dimensional (3D) biomimetic environment capable of guiding stem cells to chondrogenic differentiation. A supercritical emulsion extraction technology was used to produce the PLGA-MCs and tailored to sustain the delivery capacity into the collagen hydrogels for 21 days. The seeding of human Bone Marrow Mesenchymal Stem Cells (hBM-MSCs) into the collagen matrix together with the PLGA-MCs showed chondrogenic induction by the upregulation of specific chondrogenic markers under dynamic conditions. From histological and immunofluorescence analysis, it was confirmed the chondrogenic extracellular matrix formation. Cells immunomodulatory activity was also confirmed by proinflammatory and anti-inflammatory cytokines gene expression by the hBM-MSC under dynamic conditions. This study showed that the use of a 3D hydrogel environment combined with growth factor-controlled delivery are suitable as tissue engineered models for the study in vitro chondrogenic differentiation, which opens clinical possibilities for the use of injectable collagen-based advanced therapies.

Oliveira et al. [8], encapsulated chondroitin sulfate modified poly(amidoamine) dendrimer nanoparticles (NPs) covalently bonded to monoclonal anti-TNF α antibody, in Tyramine-Gellan Gum and Tyramine-Gellan Gum/Silk Fibroin hydrogels as two novel therapies for rheumatoid arthritis (RA) treatment. It was reported an effective NP-antibody functionalization and TNF-α capture, as well as, effective encapsulation and release of the NPs. The therapies were tested in vitro using pro-inflammatory THP-1 cells (i.e., human monocytic cell line), showing good anti-inflammatory activity in static conditions. However, in dynamic culture conditions using a dual-chamber bioreactor the THP-1 cells showed a significant reduction of TNF-α. Authors concluded that the developed approach has potential to be used as personalized medicine in RA treatment.

The structure and biocompatibility of a hydrogel based on cellulose nanofibers (CNFs) combined with alginate/pectin and enriched with 1% or 5% of 5-Fluorouracil (5-FU) were investigated by Balahura et al. [9], revealing favorable cell performance when pectin was dispersed within CNFs. Moreover, the exposure of tumor cells to CNF/5-FU induced cytotoxicity, increased levels of released caspase-1 and ROS production. Simultaneously, increased levels of p53 and caspase-1 expressions were obtained determined by the presence of 5-FU. The CNF/5-FU scaffolds inhibited breast tumor cells growth and potentially supported human adipose-derived stem cell growth, suggesting these 3D systems for soft tissue reconstruction post-mastectomy.

Lara-Ochoa et al. [10] proposed a review paper focused on the overview of the important properties of hydroxyapatite nanoparticles and their role as a drug delivery systems. Properties such as shape, size, morphology, and ionic substitution were explored, as well as, their correlation with the biological response. The main chemical composition and applications of hydroxyapatite, the benefits of using nanoparticles, and the influence of their morphology and crystallinity in biological response are explored. A special importance was attributed to the charge and chemical/physical interactions of the nanoparticles. Finally, authors discussed the tailoring of hydroxyapatite nanoparticles for specific biomolecules, i.e., proteins, peptides, drugs, and genetic material.

Marine algae are rich in biologically active compounds valuable for food industry and pharmaceutical applications. Considering this, Aboeita et al. [11] used a ultrasound-assisted extraction (UAE) method to extract carbohydrate content from the red algae, *Pterocladia capillacea*. The extract showed potent antioxidant activity, and was used as capping agent in the green synthesis of cooper nanoparticles. The produced CuO nanoparticles were subsequently loaded with nedaplatin that was sustained released up to 120 h. The formulation also showed cytotoxicity against hepatocellular carcinoma, breast cancer and ovarian cancer cell lines, demonstrating its anticancer effects.

Ribeiro et al. [12] proposed biomimetic composite tubular grafts based on a horseradish peroxidase (HRP) crosslinking method to form silk fibroin (SF) hydrogels containing ZnSr-doped β-tricalcium phosphate (ZnSr-β-TCP) particles. These were proposed as bone tunnel fillers in anterior cruciate ligament (ACL) grafts implantation approaches. The tubular structures presented homogeneous micro-structure and powder’s incorporation, good mechanical properties and crystalline conformation. Moreover, the swelling properties were suitable to fulfil the space created in the bone structure after bone tunnel enlargement, together with a stable degradation profile of the tubes. From in vitro studies, it was observed that SaOs-2 cells adhered and proliferated into the tubular grafts. Moreover, they also presented osteogenic inducement which is vital to stimulate bone tissue regeneration and faster osteointegration while connecting with ACL tissue.

The research progresses of different local drug delivery systems using titanium-based implants to promote bone–implant integration were overviewed by Meng et al. [13]. Authors reported the lack of superior osseointegration promoted by previous traditional and surface modified titanium-based implants. Nevertheless, the development of local drug delivery systems, alone or combined with traditional surface modification methods, on titanium-based implants has been shown to be effective to improve osseointegration.

Guarch-Pérez et al. [14] developed a composite of poly-ε-caprolactone, hydroxyapatite and halloysite nanotubes loaded with gentamicin sulphate to be used as bone fixation plates coatings, using a fused filament fabrication 3D printing technology. Bacterial infections are a problematic in orthopedic and trauma surgery, which was why the authors proposed a coating system to act as a local antibiotic prophylaxis providing dosage and bioavailability at the bone site with minimum toxic effects. The composite biomaterial loaded with gentamicin sulphate showed complete antimicrobial effects on *Staphylococcus aureus* in an ex vivo mouse femur fixation plate infection model. Moreover, it was possible to prevent *S. aureus* infection in the bone and surrounding tissue using an in vivo mouse model. This study, represents a newly and successful method to locally prevent bacterial infection in vivo using biomaterials. Additionally, the possibility of using fused filament fabrication 3D printing in the creation of patient-specific implants is now a reality for a wider range of personalized medicine.

Nowadays, the number of patients suffering from poorly bone healing is still high. Thus, Oude Egberink et al. [15] proposed porous collagen scaffolds incorporating peptide-mRNA nanoparticles (NPs) as functional biomaterials for applications in bone regeneration. It has been shown that messenger RNA (mRNA) constitutes a superior alternative to protein delivery (e.g., BMP-2) enabling a prolonged expression and local action. Peptide-mRNA complexes were generated in NPs and uniformly distributed throughout the scaffolds, showing preserved cell viability and attachment. Moreover, the protein expression was dependent on the incorporated amount of mRNA. Overall, it was shown that the collagen scaffolds incorporating peptide-mRNA complexes are promising as off-the-shelf biomaterials for different regenerative medicine applications.

Finally, Escobar et al., [16] showed the successful loading of glial cell line-derived neurotrophic factor (GDNF) on carboxymethyl chitosan/poly(amidoamine) (CMCht/PAMAM) dendrimer nanoparticles (NPs) for the exogenous administration of the growth factors. A high stability of the NPs was observed, as well as, a dual controlled release profile of the GDNF, which is beneficial for peripheral nerve regeneration (PNR) purposes. The NPs were also well internalized by SH-SY5Y neuronal cells helping to conduct PNR. In brief, the authors observed promising results involving NPs incorporation with growth factors and their internalization by cells involved in the regeneration of injured nerves. Hopefully, further in vivo assays will help to gain in depth knowledge on the effectiveness of the described NPs for peripheral nerve regeneration therapies.

## References

[1] Doostmohammadi M., Forootanfar H., Ramakrishna S. (2020). Regenerative medicine and drug delivery: Progress via electrospun biomaterials. Mater. Sci. Eng. C.

[2] Pacifici N., Bolandparvaz A., Lewis J.S. (2020). Stimuli-responsive biomaterials for vaccines and immunotherapeutic applications. Adv. Ther..

[3] Bordoni M., Scarian E., Rey F., Gagliardi S., Carelli S., Pansarasa O., Cereda C. (2020). Biomaterials in neurodegenerative disorders: A promising therapeutic approach. Int. J. Mol. Sci..

[4] Kim H., Kwak G., Kim K., Yoon H.Y., Kwon I.C. (2019). Theranostic designs of biomaterials for precision medicine in cancer therapy. Biomaterials.

[5] Li L., Wei X.-W. (2020). Recent advances in multifunctional nanoengineered biomaterials. Nanoeng. Biomater. Adv. Drug Deliv..

[6] Kang J., Cai Y., Wu Z., Wang S., Yuan W.-E. (2021). Self-encapsulation of biomacromolecule drugs in porous microscaffolds with aqueous two-phase systems. Pharmaceutics.

[7] Lamparelli E.P., Lovecchio J., Ciardulli M.C., Giudice V., Dale T.P., Selleri C., Forsyth N., Giordano E., Maffulli N., Della Porta G. (2021). Chondrogenic commitment of human bone marrow mesenchymal stem cells in a perfused collagen hydrogel functionalized with hTGF-β1-releasing PLGA microcarrier. Pharmaceutics.

[8] Oliveira I., Fernandes D., Maia F., Canadas R., Reis R., Oliveira J. (2021). Bioengineered nanoparticles loaded-hydrogels to target TNF Alpha in inflammatory diseases. Pharmaceutics.

[9] Balahura L.-R., Dinescu S., Balaș M., Cernencu A., Lungu A., Vlăsceanu G., Iovu H., Costache M. (2021). Cellulose nanofiber-based hydrogels embedding 5-FU promote pyroptosis activation in breast cancer cells and support human adipose-derived stem cell proliferation, opening new perspectives for breast tissue engineering. Pharmaceutics.

[10] Lara-Ochoa S., Ortega-Lara W., Guerrero-Beltrán C.E. (2021). Hydroxyapatite nanoparticles in drug delivery: Physicochemistry and applications. Pharmaceutics.

[11] Aboeita N.M., Fahmy S.A., El-Sayed M.M.H., Azzazy H.M.E.-S., Shoeib T. (2022). Enhanced anticancer activity of nedaplatin loaded onto copper nanoparticles synthesized using red algae. Pharmaceutics.

[12] Ribeiro V.P., Costa J.B., Carneiro S.M., Pina S., Veloso A.C., Reis R.L., Oliveira J.M. (2022). Bioinspired silk fibroin-based composite grafts as bone tunnel fillers for anterior cruciate ligament reconstruction. Pharmaceutics.

[13] Meng F., Yin Z., Ren X., Geng Z., Su J. (2022). Construction of local drug delivery system on titanium-based implants to improve osseointegration. Pharmaceutics.

[14] Guarch-Pérez C., Shaqour B., Riool M., Verleije B., Beyers K., Vervaet C., Cos P., Zaat S.A. (2022). 3D-printed gentamicin-releasing poly-ε-caprolactone composite prevents fracture-related staphylococcus aureus infection in mice. Pharmaceutics.

[15] Oude Egberink R., Zegelaar H.M., El Boujnouni N., Versteeg E.M., Daamen W.F., Brock R. (2022). Biomaterial-mediated protein expression induced by peptide-mRNA nanoparticles embedded in lyophilized collagen scaffolds. Pharmaceutics.

[16] Escobar A., Carvalho M.R., Maia F.R., Reis R.L., Silva T.H., Oliveira J.M. (2022). Glial cell line-derived neurotrophic factor-loaded CMCht/PAMAM dendrimer nanoparticles for peripheral nerve repair. Pharmaceutics.

